# Examining Patient Characteristics Associated With Digital Outpatient Care for Type 1 Diabetes (DigiDiaS): Cross-Sectional Study

**DOI:** 10.2196/73708

**Published:** 2025-08-22

**Authors:** Ingeborg Spildo, Heidi Holmen, Annesofie Lunde Jensen, Milada Hagen, Tone Singstad, Jacob Andreas Winther, Eirik Årsand, Astrid Torbjørnsen

**Affiliations:** 1 OsloMet – Oslo Metropolitan University Oslo Norway; 2 Aarhus University Aarhus Denmark; 3 Steno Diabetes Centers Aarhus Denmark; 4 Akershus University Hospital Lørenskog Norway; 5 UiT The Arctic University of Norway Tromsø Norway

**Keywords:** diabetes mellitus, type 1, telemedicine, remote consultation, telenursing, distance counseling, delivery of health care, patient-reported outcome measures

## Abstract

**Background:**

People with type 1 diabetes require ongoing self-management and frequent follow-up care. Digital care models might offer flexible solutions and increased sustainability*,* and while the clinical opportunities of these care models have been explored, the characteristics of patients inclined to opt for digital care remain unclear.

**Objective:**

This study aimed to investigate which selected sociodemographic and disease-related patient characteristics are associated with opting for digital outpatient care among patients with type 1 diabetes.

**Methods:**

This cross-sectional study was conducted at the endocrinology department of Akershus University Hospital in Norway, as part of a larger longitudinal study. Adult patients with type 1 diabetes were eligible to participate and were recruited consecutively. Patients could choose either a novel, mobile health–based, digital, tailored, outpatient care model (DigiDiaS care) or continuation of usual care. DigiDiaS care is delivered via an app comprising a message service; preconsultation questionnaires; options for physical, video, or telephone consultations; an information page; and an e-learning course. Sociodemographic and clinical data were collected from the participants’ medical records and the national diabetes registry. Self-reported data comprised self-management measured using the Patient Activation Measure 13 (PAM-13), diabetes distress (20*-*item Problem Areas in Diabetes; PAID-20), well-being (World Health Organization-Five Well-Being Index; WHO-5), and health literacy (12-item short version of the European Health Literacy Survey Questionnaire; HLS-19 Q12) questionnaires. We explored group differences and conducted logistic regression to identify factors associated with opting for DigiDiaS care versus usual care.

**Results:**

A total of 237 patients consented to participate in the study, with 185 (78.1%) opting for DigiDiaS care and 52 (21.9%) opting for usual care. The DigiDiaS care group had a statistically significantly shorter duration of diabetes (median 19, range 0-51 years) compared with the usual care group (median 29, range 3-58 years; *P*<.001); higher proportions of users of insulin pumps than insulin pens for insulin delivery (DigiDiaS care: 74/185, 40%; usual care: 12/185, 23.1%; *P*=.007), and a lower median well-being care score (DigiDiaS care: median score 60, range 4-96; usual care: median score 68, range 16-100; *P*=.04). The DigiDiaS care and usual care groups did not differ in sociodemographic variables, presence of late complications from diabetes or comorbidities, self-management, diabetes distress, or health literacy.

**Conclusions:**

This study reveals that most patients with type 1 diabetes choose digital outpatient care when it is offered. Our study suggests that those opting for DigiDiaS care are already familiar with using diabetes-related technology, have a shorter diabetes duration*,* and have lower well-being. It is essential to understand the characteristics of patients who opt for usual care to ensure high-quality health care services. Further studies should also focus on how the implementation of digital solutions in outpatient care can affect how and if patients use them.

**International Registered Report Identifier (IRRID):**

RR2-10.2196/52766

## Introduction

### Background

Living with type 1 diabetes can be challenging and is associated with health complications such as poor mental well-being and poor quality of life, and it requires lifelong self-management and support to prevent these complications [1-3]. A life with type 1 diabetes can affect patients’ ability to self-manage, increasing their risk of diabetes complications [4,5]. Often, diabetes care and self-management education and support are provided through multidisciplinary teams [6-8]. However, the traditional calendar-based approach in outpatient care is often rigid, giving patients little opportunity to influence their care. Consequently, the calendar-based approach threatens health care service resource sustainability and presents several challenges; it is not given that outpatient care is appropriate or necessary at the time of the consultation [7,9,10]. Conversely, a patient-centered approach in outpatient care, where patients themselves influence the timing and nature of treatment based on patients preferences and needs, has been suggested as a solution to these problems [11]. Furthermore, individualization through patient-centered care can strengthen the patient’s autonomy and participation in their own treatment.

The increasing focus on individualizing care aligns with an increased recognition of how digital solutions in outpatient care can provide closer interaction with health care services [8]. Such digital outpatient care may include various forms of synchronous and asynchronous remote patient monitoring; telephone and video consultations; messaging services; connection to wireless tools, such as glucose sensors and insulin pumps; or other forms of telecommunication tools [12]. Allowing and facilitating a more active patient role, patient-reported outcomes (PROs) can be integrated into digital tools for follow-up care [8]. PROs offer direct feedback from patients themselves about their health, which, among other things, provides a broader perspective on clinical goals and serves as a conversational tool [13,14]. While PROs in clinical care have gained increasing attention in recent years, a scoping review found the use of PRO as part of routine treatment in diabetes outpatient settings to be scarce [15].

Although interest in and recognition of the opportunities for digital outpatient care have been acknowledged by international panels of health care professionals in recent years [8], few studies have investigated PROs in digital outpatient care [15]. A pioneering study from Denmark used the PRO measure DiabetesFlex before scheduled consultations delivered in a digital solution to allow patients to voice their need for consultations or cancel if not deemed necessary [16]. This resulted in reduced face-to-face visits and improved well-being and diabetes distress while providing safe diabetes management. In addition, DiabetesFlex altered the interactions between clinicians and patients as it shifted toward the patients’ perspectives on living with diabetes [17]. It increased patient involvement, strengthened their sense of responsibility, and tailored diabetes care to meet individual needs [16,17].

Despite previous research emphasizing the positive contribution of digital outpatient care solutions in diabetes outpatient care, there remains a lack of research to establish an evidence base. For instance, not everyone wanted to continue with DiabetesFlex after the end of the study period, despite their positive experiences [17]. Furthermore, there is a lack of knowledge on the characteristics of patients choosing digital or flexible diabetes follow-ups. Characteristics such as sociodemographics, clinical measures, and disease-specific measures can reveal whether users and nonusers of digital outpatient care differ and whether they possess specific traits or factors. Exploring such characteristics can provide insights into the actions and choices of patients with type 1 diabetes in their outpatient care [18-20].

### Objective

This study aims to investigate which selected sociodemographic and disease-related patient characteristics are associated with opting for digital outpatient care among patients with type 1 diabetes.

## Methods

### Study Design

This study used a cross-sectional design to investigate characteristics of patients with type 1 diabetes opting for digital outpatient care compared with those opting for usual care. The data used in this study is collected as part of a larger multimethod prospective observational study assessing the integration of digital outpatient care procedures for patients with type 1 diabetes and their clinicians [21]. In this paper, we explored the baseline characteristics of these participants, and the recruitment of participants and data collection took place from October 2022 to October 2023. This study is reported according to the Strengthening the Reporting of Observational studies in Epidemiology (STROBE) reporting guideline [22].

### Setting

The study was carried out at the Endocrinological Outpatient Clinic at Akershus University Hospital. In November 2021, the clinic developed and introduced a voluntary digital mobile health (mHealth) supplement outpatient care program specifically tailored to patients with type 1 diabetes called DigiDiaS.

### Participants

#### Eligibility Criteria

The inclusion and exclusion criteria for participants are listed in Textbox 1. Both users and nonusers of DigiDiaS care were eligible for participation.

**Eligibility criteria.**

**Inclusion criteria**

- Aged ≥18 years
- Having type 1 diabetes and receiving treatment and care at the endocrinological outpatient clinic at Akershus University Hospital
- Ability to read and understand Norwegian

**Exclusion criteria**

- Having type 2 diabetes or gestational diabetes
- Individuals with impaired cognitive abilities who are unable to complete the self-reported data independently or understand the nature of the study

#### Recruitment

Participants were recruited through consecutive sampling. There was no randomization, and the participants could choose whether they opted for DigiDiaS care or to continue with usual care. Participants were introduced to the study and invited to participate during a routine consultation with a diabetes specialist nurse or an endocrinologist. If they were not already using the digital outpatient care DigiDiaS, they were provided with details on both the study and the digital outpatient care solution. For patients interested in DigiDiaS care and new to this digital outpatient care solution, their diabetes specialist nurse provided a brief introduction to the app and offered support to download it, if needed. The patients received brief written and oral information about the study and were asked if a researcher (IS) could contact them. Those consenting to further information received a phone call before they received a link to a digital consent form. If a patient expressed a desire to participate in the study over the phone but did not consent, a reminder was sent via text message. The written informed consent form was also made available in paper format, if needed.

### Diabetes Outpatient Care

#### Usual Care

At the endocrinology outpatient clinic in which this study was conducted, usual care for type 1 diabetes follows a routine, calendar-based system in accordance with Norwegian national diabetes guidelines [6]. In Norway, all patients with type 1 diabetes are offered follow-up care in specialist outpatient care, provided by interdisciplinary health care teams, including endocrinologists, diabetes-specific nurses, and dieticians. This care includes an extended annual check-up and a routine midyear check-up. Patients with recent diagnoses of diabetes, comorbidities, late complications, or poorly controlled diabetes may receive more frequent consultations. Under this system, appointments are approximately prescheduled, giving patients limited flexibility to influence the timing and nature of their own outpatient care. The usual care group in this study continued with this routine—calendar-based system of receiving a prescheduled time for consultation with limited flexibility to influence their follow-up care.

#### DigiDiaS Care

DigiDiaS care serves as a flexible digital supplement designed to support the patients’ interactions with clinicians during outpatient care, allowing the individual patient’s needs to guide contact with clinicians [21]. DigiDiaS care is delivered through a free app that patients can download onto their private smartphones from the App Store or Google Play, following an invitation from a health care professional. The main components in DigiDiaS care provided by Dignio Connected Care are simple and intuitive to use, comprising the patient app MyDignio and the clinician’s software DignioPrevent delivered through a web browser via a direct link from the medical record system. Dignio Connected Care meets the necessary safety requirements, including CE certification [23].

The digital outpatient care solution includes several features in the MyDignio app for patients. First, a messaging service enables patients to contact the health care service outside of planned outpatient care, if necessary, with asynchronous messages. Second, before scheduled follow-up consultations, patients receive a PRO-based questionnaire, which includes questions about well-being, diabetes-specific outcomes, and the need for a consultation and questions for patients to prepare topics they want to address in the scheduled outpatient care. The PRO-based questionnaire is based on the Danish DiabetesFlex [16]. It was adapted to Norwegian and further refined in collaboration with user representatives, clinicians, and managers to suit the digital platform used at the clinic [13]. The third feature of DigiDiaS care is that patients can choose between remote video consultation, telephone consultation, or in-person attendance for routine outpatient care consultations. Finally, clinicians can provide patients with e-learning courses and create a tailored information page for each patient, if needed. Patients receive notifications about new activities from the app through push notifications or text messages.

For clinicians, digital outpatient care is conducted through software accessible from web app DignioPrevent, providing clinicians with features for flexible patient care [21]. Through this system, clinicians manage the asynchronous chat, send out the PRO-based questionnaire before consultations, and edit the information page. The answers on the PRO-based questionnaires have defined thresholds dependent on the patients’ responses, using a traffic light model, with green, yellow, and red alerts and an additional orange alert if the questionnaire has not been completed. Green questionnaires indicate no issues and are not visible in DignioPrevent; however, all yellow and red questionnaires from the past are available for comparison. Clinicians also provide patients with information and e-learning courses, both through premade templates and an open-text field. It is also possible to contact the patient directly through video consultation with DignioPrevent. However, for scheduled appointments, a separate system called Whereby is used for video consultations as it communicates with the calendar within the medical record system that DignioPrevent does not.

### Data Collection

Data were collected through 3 primary sources: self-reported questionnaires, electronic medical records, and the national quality registry for diabetes, the Norwegian Organization for Quality Improvement of Laboratory Examinations (NOKLUS). Self-reported data and the digital consent form were collected using Nettskjema [24], a secure platform that stores data in the Services for Sensitive Data (TSD) platform for confidential handling. To deliver the digital consent form and self-reported data, the patients received a secure link via SMS text message or email, requiring them to log in with their national ID for secure access. Patients who preferred the consent form and self-reported data on paper received this and submitted these papers back to the clinic which was stored in a locked safety box. Data from medical records and the national diabetes quality registry (NOKLUS) were manually extracted into Microsoft Excel files, stored, and managed in TSD. To ensure the accuracy of the data extraction, a coresearcher cross-checked all extracted data against the original medical records and NOKLUS registry, as described in the protocol [15].

In addition, to compare our sample with the overall hospital sample comprising all patients with type 1 diabetes and the national population of people with type 1 diabetes, we received aggregated data directly from NOKLUS on the variables age, gender, education, years since diabetes duration, insulin delivery method, and glycated hemoglobin (HbA_1c_), all provided in median and range. Comparing our sample with the national population is essential to identify and assess biases, ensuring that our findings are representative and applicable to the broader hospital sample and national population.

To explore the role of patients’ travel time from home to the outpatient clinic, an estimate of travel time was calculated based on the patients’ postal code. Google Maps was used to determine the travel time by car to the main premises of the hospital. Akershus University Hospital is a local hospital with a defined coverage area. Participants living outside this area were typically students or recent movers who had not switched to their local hospital or individuals who, for other reasons, did not receive outpatient care at their local hospital. Public transport is only available for parts of the coverage area and was therefore not used in the calculation.

### Measures

#### Sociodemographic and Clinical Variables

Sociodemographic variables, gathered from the medical records, included age, gender, education, employment, ethnicity, comorbidities, cohabitation, single resident status, travel time to the hospital, and use of other health-related applications (Table 1). Clinical variables comprised diabetes duration, HbA_1c_, insulin delivery method, blood glucose monitoring method, time-in-range, diabetic ketoacidosis, hypoglycemia, late complications from diabetes, BMI, cholesterol, blood pressure, and comorbidities (Table 2). While time-in-range can be provided in various ranges when aiming to present the ambulatory glucose profiles of the patients, in this study, we present time-in-range for the last 14 days as this is most frequently used in this study’s clinical setting. All clinical variables are from the last measurement before consent to participate in the study, except for HbA_1c_, which is the last measurement inclusion in the digital solution for the DigiDiaS care group, and the last measurement before consent to the study for the usual care group. Self-reported data encompassed self-management, diabetes distress, well-being, and health literacy (Table 3).

**Table 1:** Sociodemographic variables.

Variables	Categories or data type	Merged into categories for analysis	Data source
Age	Years	—^a^	Medical records
Gender	Woman or man	—	Medical records
Education	Not completed primary school (10 y), primary upper secondary school (13 y), vocational school (13 y), college or university (≤4 y), college or university (>4 y), unknown	“13 years or less” and “over 14 years”	Medical records
Employment status	Full- or part-time employment, being a student, job seeking, receiving disability benefits, and retirement (based on the Norwegian retirement age of 67 y)	—	Self-reported
Ethnicity	European, Asian, African, or unknown	European, Asian, or African, with a separate category for missing data	Medical records
Cohabitation	Living in a partnership or not living in a partnership	Yes or no	Medical records
Single resident	Living alone or not living alone	Yes or no	Medical records
Travel time to hospital	Estimate based on postal code using Google Maps to identify travel time by car, in min	>30 min travel time, <30 min, or living outside of coverage area	Medical records and Google Maps
Use of other health-related apps	One question with the response alternatives “daily” “weekly,” “monthly,” “not using this now but having used it before.” or “not using other health-related apps”	Daily or weekly use, monthly use or used before, never or previous use	Self-reported

^a^Not applicable.

**Table 2:** Clinical variables.

Variables	Categories or data type	Merged into categories for analysis	Data source
Diabetes duration	The year from onset to when participants consented to participate in this study	—^a^	NOKLUS^b^ registry
HbA_1c_^c^<75 mmol/mol	HbA1c (mmol/mol) on a continuous scale The proportion of participants with HbA1c above 75 mmol/L	—	NOKLUS registry
Insulin delivery	Pump or pen	—	NOKLUS registry
Blood glucose monitoring	CGMd or glucometer	—	NOKLUS registry
Time-in-range, for those using CGM	Percentage of time-in-range (3.9-10 mmol/L) last 14 d	—	NOKLUS registry
Diabetic ketoacidosis	Never Once Several times or unknown	Recoded into none, 1≥, missing	NOKLUS registry
Hypoglycemia	Never Once Several times or unknown	Recoded into none, ≥1, missing	NOKLUS registry
Late complications from diabetes	Albuminuria Dialysis treatment Kidney transplantation Retinopathy Neuropathy Stroke Arterial vascular surgery Amputation Diabetic foot ulcers	Recoded into none, 1, ≥2, and missing	NOKLUS registry
BMI (kg/m^2^)	Weight and height	BMI=weight (kg)/[height (m)]^^^2	NOKLUS registry
Cholesterol	Low-density lipoprotein	—	NOKLUS registry
Blood pressure	Systolic blood pressure and Diastolic blood pressure in mm Hg	—	NOKLUS registry
Comorbidities	Charlson Comorbidity Index, which includes a range of conditions such as myocardial infarction, congestive heart failure, peripheral vascular disease, and others	None, 1, ≥2, and missing	Self-reported

^a^Not applicable.

^b^NOKLUS: Norwegian Organization for Quality Improvement of Laboratory Examinations.

^c^HbA_1c_: glycated hemoglobin.

^d^CGM: continuous glucose monitoring.

**Table 3:** Measures for self-management, diabetes distress, well-being, and health literacy.

Measures	Items, scale, and domains	Interpretation
Self-management (PAM-13^a^)	13 items, rated from 1 “disagree strongly,” to 4, “strongly agree,” with an additional “not applicable” 4 domains: knowledge, beliefs, confidence, and skills for managing one’s health.	The total PAM-13 score is calculated using a formula that weighs the various questions and that forms a scale from 0-100. Higher scores indicate higher activation [25]. A 3.2 change on a 1-100 scale is considered small but clinically relevant [26] There are 4 levels: Level 1 (score ≤47), disengaged and overwhelmed Level 2 (score 47.1-55.1), becoming aware but still struggling Level 3 (score 55.2-67), taking action Level 4 (score≥67.1), maintaining behavior and pushing further Levels 1 and 2, 3 and 4 were merged into categories for analysis
Diabetes distress (PAID^b^)	20 items, ranked on a 5-point Likert scale from 0, “not a problem” to 4, “serious problem”	Sum of all 20 items and multiplied by 1.25, giving a total score ranging from 0-100 [26] A higher score reflects greater emotional distress. A score of ≥40 indicates severe emotional distress.
Well-being (WHO-5^c^)	The 5 items are measured in a 6-point Likert scale from 5 "all the time" to 0 "at no time".	Sum of 5 items multiplied by 4 to create a scale from 0-100 [27] A higher score indicates higher well-being Score <50 may be an identification of mild to severe depressive affect [28]
Health literacy (HLS19-Q12^d^)	The 12 items are measured in a 4-point Likert scale from 1, “very hard” to 4, “very easy” with added “I don’t know”	Sum of the 12 items is calculated. Higher scores reflect higher health literacy [29] The scale is divided into 4 levels with expected characteristics: Below level 1; lack key knowledge and skills about health and may have challenges in understanding and applying health information Level 1; where you are expected to be able to access, understand, and apply health information relevant to staying healthy Level 2; access, appraise, understand, and apply health information and advice relevant to enhancing physical and mental health Level 3; access, appraise, understand, and apply health information and advice relevant to making informed health care choices by critically evaluating health claims and judiciously comparing treatments The items can also be distributed into the 3 different domains they measure—health care, disease prevention, and health promotion, and the individual domain can be scored with a minimum of points 0 maximum of 16 points per domain

^a^PAM-13: Patient Activation Measure 13.

^b^PAID: Problem Areas in Diabetes.

^c^WHO-5: World Health Organization-Five Well-Being Index.

^d^HLS19-Q12: 12-item short version of the European Health Literacy Survey Questionnaire.

#### Self-Management

Self-management and the ability to manage one’s health was assessed with the “Patient activation measure” short version (Patient Activation Measure 13 [PAM-13]) [30] (Table 3). Hibbard et al [30,31] developed and validated the PAM-13 questionnaire as a generic questionnaire for people with and without chronic conditions. The PAM-13 questionnaire has been validated in Norwegian and has previously been used to evaluate patient education in various diagnoses, including type 2 diabetes [25,32,33]. It has been used in multiple contexts across several countries, with findings suggesting that Norwegians generally exhibit higher activation than other populations [33]. Furthermore, the PAM is also suitable for the evaluation of digital interventions [34]. The reliability of the PAM-13 scale in this study was confirmed with a Cronbach α of 0.894, indicating high internal consistency among the items.

#### Diabetes Distress

Diabetes distress was assessed using the disease-specific questionnaire 20-item Problem Area in Diabetes (PAID-20) [26] (Table 3). PAID-20 has been previously translated and validated in Norwegian by Graue et al [35] and has been used as a tool to prepare for consultations for patients with type 1 diabetes in a previous study [36]. The reliability of the PAID-20 scale in this study was confirmed with a Cronbach α of 0.939, indicating excellent internal consistency among the items.

#### Well-Being

Psychological well-being and quality of life were measured with the generic tool World Health Organization–Five Well-Being Index (WHO-5) [37] (Table 3). WHO-5 has been translated to Norwegian and is validated for the type 1 diabetes population [27,38,39]. A score <50 may indicate that the patient is feeling unwell and should be examined further to investigate mild to moderate depression [40]. The reliability of the WHO-5 in this study was confirmed with a Cronbach α of 0.826, indicating high internal consistency among the items.

#### Health Literacy

Health literacy and the ability to make informed health choices were measured using the 12-item short version of the European Health Literacy Survey Questionnaire (HLS19-Q12) [41]. HLS_19_-Q12 is translated into many languages and has been used in 19 European countries, including Norway [42] (Table 3). The HSL_19_-Q12 has been validated and used in both a large general population and in patients with type 2 diabetes in Norway [41,43,44]. The reliability of the HLS_19_-Q12 scale in this study was confirmed with a Cronbach α of 0.871, indicating high internal consistency among the items.

### Ethical Considerations

As this research project involves human subjects, the project has been reported to the Norwegian Agency for Shared Services in Education and Research (SIKT; reference number: 456954) and approved by the local data protection officer at Akershus University Hospital (reference: 2022_125). The project was presented to the Regional Committees for Medical and Health Research Ethics (REK; reference number: 407539), and they deemed it to be outside their scope and defined as a quality improvement project.

All participants received oral and written information before providing informed consent to participate in this study and filling out the self-reported data. Digital consent forms, medical records data, and self-reported data were stored and managed in TSD. Participants provided their social security numbers for in the consent form but were assigned ID numbers for deidentification. The code key was stored in a separate file within TSD. Data received from NOKLUS for comparison with the overall hospital sample and the national population were aggregated and thus anonymous. The study participants were not compensated for participating in this study.

### Statistical Analysis

Categorical variables are described with counts and percentages; continuous variables are presented with median values and ranges. To compare DigiDiaS care and usual care, we used nonparametric chi-square tests for pairs of categorical variables and Mann-Whitney *U* tests for pairs of continuous data. Missing data were not imputed, and the counts and proportions of missing values for each variable are presented in tables. Responses such as “don’t know” or “not applicable” in self-reported questionnaires were treated as missing values. To assess internal consistency of the scales used in the study, Cronbach α was calculated for the validated questionnaires [45].

To assess the strength of the possible association between the selected patient- and disease-related variables and the outcome, univariate logistic regression was conducted. Variables that were statistically significant in univariate analyses were entered into a multiple regression model, and the final model was fitted using the backward conditional selection process. The results are expressed as odds ratios (ORs) with 95% CIs. All tests were 2-sided, and *P* values <.05 were considered statistically significant.

In addition, to compare our sample with the overall hospital sample comprising all patients with type 1 diabetes and the national population of people with type 1 diabetes, we calculated CIs for the median values in our study sample to compare with the median and min-max values of the hospital sample and national population.

All data were processed using Excel (Microsoft Office Professional Plus 2016) and SPSS (version 29.0.0.0).

## Results

### Overview

**Figure 1:**
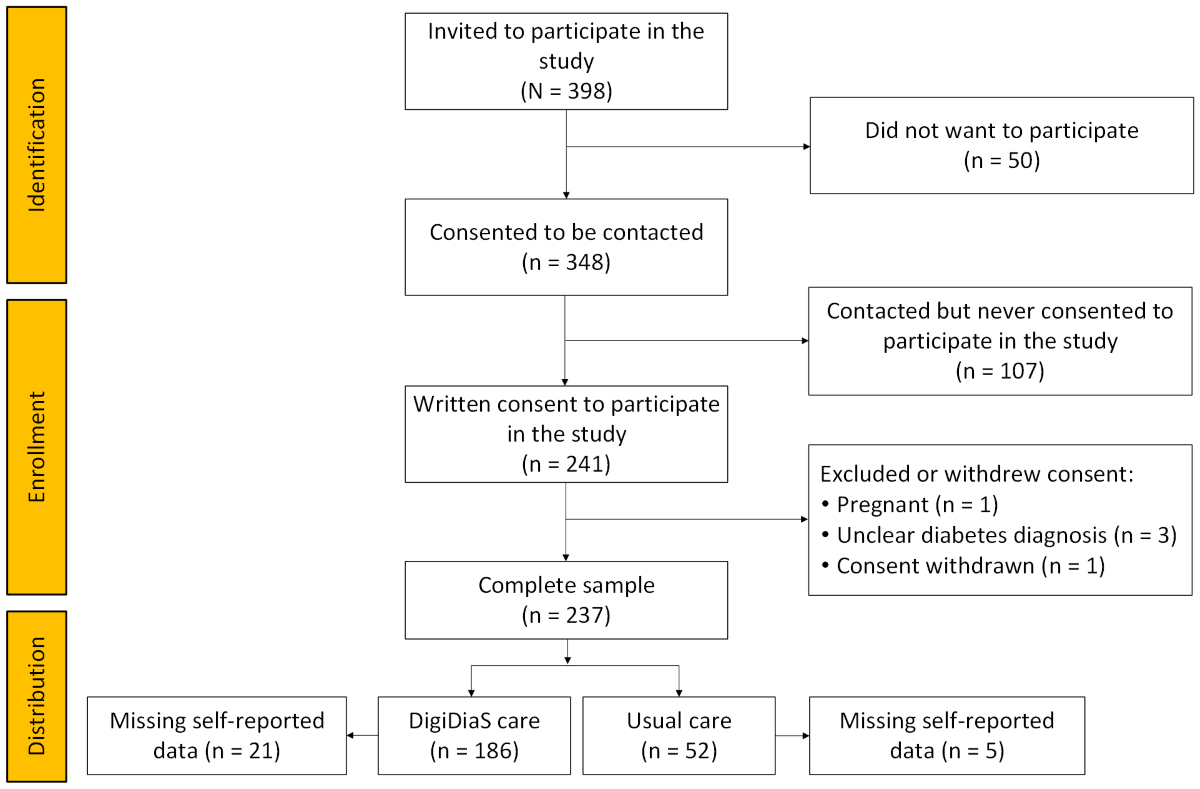
Flowchart of the participants using DigiDiaS care and usual care.

Of the 398 patients invited to participate in the study, 241 consented to participate (Figure 1). Of the 237 patients included, 185 patients opted for DigiDiaS care, and 52 patients opted to continue with usual care. In the DigiDiaS care groups, 21 self-reported data were missing and 5 from the usual care group.

#### Demographics

In the complete sample, the included participants ranged in age from 19 to 81 years, with a median age of 49 years (Table 4). The median duration of diabetes was 21 (range 0-58; Table 5) years. Overall, one-third of the participants had university or college education at the bachelor level or higher (81/237, 34%), and the majority were either currently employed or students (139/237, 58.6%; Table 4). Just under half of the participants had an estimated travel time to the hospital of >30 minutes (98/237, 41.2%). Over half of all participants used other health-related apps daily (137/237, 57.8%).

**Table 4:** Demographic variables.

Demographic variables		Total (N=237)	DigiDiaS care (n=185)	Usual care (n=52)	*P* value	
Age (y), median (range)		49 (19-81)	48 (19-79)	59 (23-81)	<.001	
**Gender, n (%)**						
	Women	108 (45.8)	91 (49.2)	17 (32.7)	.04	
**Education, n (%)**						.41
	Education up to upper secondary school or vocational school (13 y)	87 (36.7)	70 (37.8)	17 (32.7)		
	University or college education up to 4 y	49 (20.7)	41 (22.2)	8 (15.4)		
	University or college education >4 y	32 (13.5)	23 (12.4)	9 (17.3)		
	Missing	69 (29.1)	51 (27.6)	18 (34.6)		
**Employment, n (%)**						.01
	Yes	139 (58.6)	114 (61.6)	25 (48)		
	No	44 (18.5)	28 (15.1)	16 (30.7)		
	Retired^a^	28 (11.8)	19 (10.2)	8 (15.3)		
	Missing	26 (10.9)	21 (11.3)	5 (9.6)		
**Ethnic origin, n (%)**						—^b^
	European	216 (91.1)	166 (89.7)	50 (96.2)		
	Asian or African	8 (3.3)	8 (4.3)	0 (0)		
	Missing	12 (5.5)	11 (5.9)	2 (3.8)		
**Cohabitation, n (%)**						.45
	Yes	148 (62.4)	117 (63.2)	31 (59.6)		
	Missing	22 (9.3)	18 (9.7)	4 (7.7)		
**Single resident, n (%)**						
	Yes	42 (17.6)	29 (15.7)	13 (25)	.12	
**Travel time to hospital by car, median (range)**		27.5 (3-73)	27.5 (3-75)	26.5 (3-60)	.75	
	Travel time >30 min, n (%)	98 (41.2)	77 (41.4)	21 (40.4)	.87	
	Living outside of coverage area^c^, n (%)	28 (11.8)	22 (11.8)	6 (11.5)	—	
**Use of other health apps, n (%)**						.27
	Daily or weekly use	137 (57.8)	111 (67.6)	26 (55.3)		
	Monthly use	50 (21.1)	39 (23.7)	11 (23.4)		
	Never or previous use	24 (11.3)	14 (8.5)	10 (21.2)		

^a^Not included in analysis due to filling the test assumption.

^b^Not applicable; cannot be computed due to small numbers.

^c^Do not have a postal code in the area where Akershus University Hospital is the local hospital.

**Table 5:** Diabetes treatment, complications, and clinical variables.

			Total (N=237)		DigiDiaS care (n=185)		Usual care (n=52)		*P* value	
Diabetes duration (y), median (range)			21 (0-58)		19 (0-51)		29 (3-58)		<.001	
**HbA_1c_ mmol/mol), median (range)**			58 (35-117)		59 (35-117)		52.5 (41-90)		.004	
	Missing, n (%)	2 (0.8)		1 (0.5)		1 (1.9)		—^a^		
HbA_1c_ >75 mmol/mol, n (%)			36 (15.2)		32 (17.3)		4 (7.7)		—	
**Insulin delivery (pump), n (%)**			86 (36.3)		74 (40)		13 (23.1)		.02	
	Missing	1 (0.4)		1 (0.5)		0 (0)		—		
**Blood glucose monitoring (continuous glucose monitoring), n (%)**			225 (94.9)		178 (96.2)		47 (90.4)		.05	
	Missing	1 (0.4)		1 (0.5)		0 (0)		—		
Time-in-range in percentage^b^, median (range)			62 (0-99)		61 (0-98)		68 (7-99)		.21	
**Diabetic ketoacidosis, n (%)**										
	≥1^c^	39 (16.5)		37 (20)		2 (3.8)		—		
	Missing	41 (17.3)		34 (18.4)		7 (13.5)		—		
**Hypoglycemia requiring assistance, n (%)**										
	≥1^c^	82 (35)		65 (35.1)		18 (34.6)		.91		
	Missing	33 (13.9)		26 (14)		7 (13.5)		—		
**Late complications from diabetes, n (%)**										.76
	None^d^	136 (57.4)		107 (57.8)		29 (55.8)				
	1^d^	63 (26.5)		51 (27.4)		12 (23.1)				
	≥2^d^	38 (16)		27 (15.5)		11 (21.1)				
	Missing	18 (7.6)		13 (7)		5 (9.6)				
**BMI (kg/m^2^), median (range)**			26.5 (16.9-43.9)		26.7 (16.9-43.9)		26.4 (19.4-42.7)		.71	
	Missing, n (%)	4 (1.7)		3 (1.6)		1 (1.9)		—		
**Low-density lipoprotein cholesterol (mmol/L), median (range)**			2.4 (0.9-5.6)		2.4 (0.9-5.6)		2.1 (0.9-3.4)		.71	
	Missing, n (%)	2 (0.8)		2 (1.1)		0 (0)		—		
**Blood pressure, median (range)**										
	Systolic blood pressure (mm Hg)	132.5 (94-178)		132 (94-178)		132 (107-166)		.28		
	Diastolic blood pressure (mm Hg)	79 (55-106)		80 (55-103)		77 (63-106)		.12		
	Missing, n (%)	18 (7.6)		13 (7)		5 (9.6)		—		
**Comorbidities, n (%)**										.62
	None	97 (40.9)		78 (42.2)		19 (36.5)				
	1	77 (32.5)		59 (31.8)		18 (34.6)				
	≥2	37 (19.7)		27 (14.5)		10 (19.2)				
	Missing	26 (11)		21 (11.4)		5 (9.6)		—		

^a^ The Norwegian retirement age is 67 years.

^b^For continuous glucose monitoring users only.

^c^One or more times.

^d^Merged into categories for analysis.

#### Clinical Variables

The median HbA_1c_ for the complete sample was 58 mmol/mol (range 35-117 mmol/mol; Table 5). Furthermore, 15.5% (36/237) had an HbA_1c_ >75 mmol/mol, considered poorly regulated. Over one-third of the participants used an insulin pump (87/237, 36.7%), while the remaining participants used an insulin pen (150/237, 63.2%) for insulin delivery. Over half of the participants had no late complications of diabetes (136/237, 57.4%), and nearly half reported no comorbidities (97/237, 40.8%).

#### Self-Management, Diabetes Distress, Well-Being, and Health Literacy

The median self-management score of 72.5 (range 9-100) corresponds with a high self-management level (Table 6). This high median score corresponds with the highest PAM level: “Maintaining behaviors and pushing forward.” Regarding the distribution across the 4 levels, half of the participants were at level 4 (116/237, 54.9%), and one-third were at level 3 for “taking action” (68/237, 32.2%).

**Table 6:** Self-reported outcomes for self-management, problem areas in diabetes, well-being, and health literacy.

		Total (n=211)	DigiDiaS care (n=164)	Usual care (n=47)	*P* value	
**Self-management (PAM-13^a^) score, median (range)**		72.5 (9-100)	72.5 (9-100)	75 (27-100)	.33	
	Level 1: disengaged and overwhelmed, n (%)	14 (5.9)	11 (5.9)	3 (5.8)		
	Level 2: becoming aware, but still struggling, n (%)	13 (5.5)	8 (4.3)	5 (9.6)		
	Level 3: taking action, n (%)	68 (28.6)	59 (31.7)	9 (17.3)		
	Level 4: maintaining behaviors and pushing forward, n (%)	116 (48.7)	86 (46.2)	30 (57.7)	.33^e^	
**Diabetes distress (PAID** ^b^ **) score, median (range)**		23.75 (0-71.25)	23.75 (0-71.25)	21.25 (0-50)	.25	
	>40, n (%)	42 (17.6)	35 (18.8)	7 (13.5)	.33	
**Well-being (WHO-5^c^) score, median (range)**		60 (4-100)	60 (4-96)	68 (16-100)	.01	
	<50, n (%)	46 (21.8)	40 (21.5)	6 (11.5)	.09	
**Health literacy (HLS19-Q12^d^) score, median (range)**		35 (13-48)	35 (13-48)	34 (19-45)	.85	
	Below level 1, n (%)	27 (12.8)	23 (14)	4 (8.5)		
	Level 1: access, understand, and apply, n (%)	52 (24.6)	38 (23.2)	14 (29.8)		
	Level 2: appraise, understand, and apply, n (%)	79 (37.4)	62 (37.8)	17 (36.2)		
	Level 3: making informed choices by critically evaluating, n (%)	53 (25.1)	41 (25)	12 (25.5)	.67^e^	
**Health literacy (HLS19-Q12) domains,** **median (range)**						.63
	Health care	12 (2-16)	12 (2-16)	12 (5-15)		
	Disease prevention	11 (3-16)	11 (3-16)	11 (4-15)		
	Health promotion	12 (4-16)	12 (4-16)	12 (5-16)		

^a^PAM-13: Patient Activation Measure; the score ranges from 0 to 100, with higher scores representing higher activation. Level 1: ≤47; level 2: 47.1 to 55.1; level 3: 55.2 to 67; level 4: ≥67.1.

^b^PAID: 20-item Problem Area in Diabetes; the score ranges from 0 to 100, with a higher score reflecting greater emotional distress. A score of ≥40 indicates severe emotional distress.

^c^WHO-5: World Health Organization-Five Well-Being Index; the score ranges from 0 to 100 with a higher score indicating higher well-being. A score <50 may be an identification of mild to severe depressive affects.

^d^HLS19-Q12: 12-item short version of the European Health Literacy Survey Questionnaire. Higher scores reflect higher health literacy. Below level 1: <27; level 1: ≥27; level 2: ≥33; and level 3: ≥39 (with associated characteristics). Domains can have a minimum score of 0 and a maximum score of 16.

^e^The *P* value represents the differences across all levels combined, not between individual levels. The level of diabetes distress was low, with a median score of 23 (range 0-71.25) of a possible maximum score of 100. However, almost 1 in 5 (42/237, 17.6%) of the participants reported scores >40, indicating diabetes distress.

Regarding well-being, the participants had a median score of 60 (range 4-100). However, 21.8% (46/237) of the participants had a WHO-5 score <50, indicating symptoms of mild to severe depression.

Overall, the participants had a fairly good health literacy score with a median health literacy score of 35 (range 13-48), characterized by the ability to appraise, understand, and apply health information (level 2).

#### Study Sample, Hospital Sample, and National Population

When comparing the study participants with the hospital sample of patients with type 1 diabetes at Akershus University Hospital, which includes all individuals with type 1 diabetes followed up at Akershus University Hospital, and the national population of patients with type 1 diabetes, which includes all individuals with type 1 diabetes in Norway, the 3 samples were comparable regarding gender, education, insulin administration, HbA_1c_, age, and diabetes duration (Table 7). However, regarding HbA_1c_ ≥75 mmol/mol, the proportion of such individuals was significantly lower in the national population compared with the study sample, indicating that our sample comprised individuals with less well-regulated diabetes.

**Table 7:** Comparison of study sample, hospital sample, and national population on sociodemographic and clinical variables.

Variables		Study sample (N=237)	Hospital sample (n=1643)	National population in Norway (n=22,408)
**Age (y; 95% CI** **46-53** **), median (range)**		49 (19-81)	46 (18-98)	47 (18-98)
	Missing, n (%)	0 (0)	0 (0)	0 (0)
**Gender, n (%)**				
	Women (95% CI 39.1-52.1)	108 (45.8)	698 (42.5)	9877 (44.1)
	Men (95% CI 47.4-60.4)	128 (54.8)	945 (57.5)	12,531 (55.9)
	Other or missing	0 (0)	0 (0)	0 (0)
**Education, n (%)**				
	Education up to upper secondary school or vocational school (13 y; 95% CI 30.5-43.1)	87 (36.7)	625 (38)	8054 (35.9)
	University or college education up to 4 y (95% CI 15.7-26.3)	49 (20.7)	268 (16.3)	4089 (18.2)
	University or college education >4 y (95% CI 9.4-18.5)	32 (13.5)	168 (10.2)	2487 (11.1)
	Missing	69 (29.1)	582 (35.4)	7778 (34.7)
**Years with diabetes (95% CI** **18-23** **), median (range)**		21 (0-58)	18 (0-78)	20 (0-78)
	Missing, n (%)	0 (0)	9 (0.5)	107 (0.5)
**HbA_1c_^a^**				
	HbA_1c_ (≥75 mmol/mol; 95% CI 10.8-20.4), n (%)	36 (15.2)	190 (12.3)	2076 (9.5)
	HbA_1c_ (mmol/mol; 95% CI 55-59), median (range)	58 (35-117)	58 (21-159)	56 (21-159)
	Missing, n (%)	2 (0.8)	93 (5.6)	667 (3)
**Insulin delivery, n (%)**				
	Pump (95% CI 30.1-42.7)	86 (36.3)	520 (31.6)	8911 (39.8)
	Pen (95% CI 57.2-69.8)	151 (63.7)	1103 (67.1)	12,960 (57.8)
	Missing	1 (0.4)	20 (1.2)	537 (2.4)

^a^HbA_1c_: glycated hemoglobin.

### Comparing Patient Characteristics in DigiDias Care Versus Usual Care

#### Demographic Variables

Most participants opted for DigiDiaS care (185/237, 78.1%), while the remaining participants (52/237, 21.9%) opted to continue with usual care. Among those choosing DigiDiaS care, half had been included in DigiDiaS care more than 90 days before consenting to participate in the study (92/185, 49.7%), thereby having had access to the digital outpatient care solution from before.

The participants in the DigiDiaS care group were significantly younger, with a median age of 47 (range 19-79) years, compared with those choosing usual care (median age 59, range 23-81 years; *P*<.001; Table 4). DigiDiaS care patients have had diabetes for a median of 10 years shorter than the usual care group (*P*<.001; Table 5). A statistically significant linear relationship was found between age and duration of diabetes in the complete sample (*P*<.001).

There was no statistically significant difference in the education level between DigiDiaS care and usual care (*P=*.42). However, there was a statistically significant higher proportion of individuals in the usual care group (16/52, 31%) who were outside the labor market or received disability benefits of working age than with DigiDiaS care (28/185, 15.1%; *P*=.01). A higher proportion lived alone and were single residents in usual care (13/52, 25%) than in DigiDiaS care (29/185, 15.7%). However, the difference was not statistically significant (*P*=.12). Further demographic variables are summarized in Table 4.

Concerning the use of other health-related mobile apps, there were more individuals in the usual care group who had never or only previously used other health-related apps (10/52, 21%) compared with the participants in the DigiDiaS care group (14/185, 8.5%); however, the difference was not statistically significant (*P*=.27; Table 4).

#### Clinical Variables

In the DigiDiaS care group, the participants had a median HbA_1c_ significantly higher at 59 (range 35-117) mmol/mol compared with 52 (range 41-90) mmol/mol in the usual care group (*P=*.004; Table 5). In terms of insulin delivery, there was a significantly higher use of an insulin pump in the DigiDiaS care group (74/185, 40%) than in the usual care group (23/52, 23%; *P=*.02).

There were no statistically significant differences in late complications from diabetes (*P=*.76). Concerning comorbidities, there were no differences between the DigiDiaS care group and usual care (*P=*.63). Further details on between-group differences in diabetes clinical variables are presented in Table 5.

#### Self-Management, Diabetes Distress, Well-Being, and Health Literacy Across the Two Groups

There were no differences between the DigiDiaS participants compared with usual care in self-management (*P*=.33), diabetes distress (*P*=.25), or the health literacy scale (*P*=.85; Table 6).

### Well-Being

The usual care group (median 68, range 16-100) had a statistically significant higher well-being score than the DigiDiaS care group (*P*=.01) indicating better well-being, with the usual care group having a higher well-being score than DigiDiaS care (median 60, range 4-96; Table 6).

Furthermore, we assessed the correlation between well-being scores and HbA_1c_ levels using Spearman correlation, finding no statistically significant relationship in the study sample, within the DigiDiaS care group (*P*=.37), or the usual care group (*P*=.33).

If the median value or percentage for the hospital selection and national population falls within the CI of the study sample, there is no significant difference. However, if the median value or percentage falls outside the CI, the groups may be significantly different.

### Group Comparison

#### Univariate Analyses

In the univariate logistic regression analyses, we identified statistically significant differences between those who chose DigiDiaS care and usual care regarding the following variables: gender, employment, diabetes duration, HbA_1c_, insulin delivery method, and well-being, as seen in Table 8. However, age was not associated with the choice of DigiDiaS or usual care.

**Table 8:** Logistic regression univariate and multivariate of the significant outcomes from Tables 2-4.

Variables	Unadjusted odds ratio (95% CI)	*P* value	Adjusted odds ratio (95% CI)	*P* value
Age (y)	0.99 (0.98-1.01)	.94	—^a^	—
Gender: man or woman (ref)	1.93 (1.01-3.70)	.04	1.10 (0.52-2.36)	.97
Employment: yes (ref) and no	2.60 (1.22-5.52)	.01	2.1 (0.94-5.07)	.68
Diabetes duration (y)	0.96 (0.93-0.98)	<.001	0.95 (0.93-0.97)	<.001
HbA_1c_	1.03 (1.00-1.06)	.01	1.02 (0.99-1.05)	.07
Insulin delivery: pen and pump (ref)	0.32 (1.09-4.52)	.02	3.16 (1.37-7.32)	.007
Well-being (WHO-5^b^ score)	0.97 (0.95-0.99)	.01	0.97 (0.95-0.99)	.03

^a^Not included in the multivariate analysis because it was not statistically significant in the univariate analysis.

^b^WHO-5: World Health Organization-Five Well-Being Index.

#### Multivariate Analysis

In multiple analyses with variables that were statistically significant in univariate analyses (Table 8), we found that a longer duration of diabetes was associated with statistically significant lower odds of opting for DigiDiaS care (OR 0.95, 95% CI 0.93-0.97). Similarly, a higher well-being score was associated with lower odds of choosing DigiDiaS care (OR 0.97, 95% CI 0.95-099). Participants using an insulin pump for insulin delivery were found to have 3 times higher odds of choosing DigiDiaS care (OR 3.16, 95% CI 1.37-7.32) in comparison to insulin pump users. Furthermore, the insulin delivery method was the variable with the strongest association with choosing DigiDiaS care versus usual care.

The multivariate logistic regression is divided into 2 rounds to avoid overfitting of the model, marked with the hard line in between—round 1 consists gender, employment in working age, and diabetes duration and round 2 consists HbA_1c_, insulin delivery, and well-being.

## Discussion

### Principal Findings

This cross-sectional study was designed to explore the characteristics of patients with type 1 diabetes who were given a choice between a novel, flexible digital mHealth supplement for outpatient care, referred to as DigiDiaS care, or continuing with their usual care.

Our main findings reveal, as expected, that the vast majority of the 238 participants included in the study opted for DigiDiaS care rather than usual care. When comparing the two groups, the DigiDiaS care group had a shorter duration of diabetes, more use of insulin pumps than insulin pens for insulin delivery, and a lower well-being score compared with the usual care group. Those who opted for DigiDiaS care did not differ from those who continued with their usual care in terms of sociodemographic variables, presence of late complications or comorbidities from diabetes, self-management, diabetes distress, or health literacy. Furthermore, our study sample did not differ from the outpatient sample or the national population on relevant variables.

### Comparison With Prior Evidence

Not surprisingly, most participants opted for DigiDiaS care, aligning well with their profile of being both younger and already users of technology for insulin administration. Using an insulin pump requires certain technical skills, which are also necessary to use the DigiDiaS care app. Using an insulin pump instead of an insulin pen was the strongest characteristic associated with the choice of DigiDiaS care over usual care. This aligns well with previous research findings on how familiarity with diabetes-related technology might drive the adoption of other digital health tools [46,47]. These findings indicate that patients already accustomed to using advanced diabetes management technologies are more likely to embrace new digital health solutions, such as DigiDiaS care. In general, patients in the usual care group had longer diabetes duration compared with those in the DigiDiaS care group, and they may have established a routine for their outpatient care, making them less inclined to try out something new. Thus, when introducing digital care solutions in diabetes management, those already using diabetes technology equipment may be more prone to exploring the possibilities within new ways of receiving outpatient care.

In our sample, those opting for DigiDiaS care had lower well-being scores than those in the usual care group. Living with a chronic condition requiring ongoing self-management, such as type 1 diabetes, can affect well-being, even though well-being can vary throughout life and with age [4]. This variation in well-being is also observed in the Norwegian general population, where well-being appears to increase with age from the early 40s to the mid-70s [48]. This might correspond with how our participants opting for DigiDiaS care had a median age of 48 years—that is, 11 years younger than those opting for usual care and reporting a higher well-being score. Compared with the Norwegian population of adults with type 1 diabetes, both groups in our sample had a median well-being score higher than the national average [49]. Thus, despite our DigiDiaS group presenting a lower well-being score compared with those continuing with usual care, all participants had a fair level of well-being without symptoms of depression interfering with their abilities to self-manage.

Although most of the recruited participants opted for DigiDiaS care, a small proportion of the participants chose to continue with usual care, thus declining the opportunity to explore digital follow-up. In our study, we did not investigate actual use but rather the willingness to use DigiDiaS care. To the best of our knowledge, DigiDiaS care is the first outpatient care model comprising specific components, such as a message service, PRO-based questionnaires, an information page, and video consultations for diabetes care [15]. The closest alternative to our knowledge is DiabetesFlex, and our study is a response to the call by Jensen et al [17] after interviewing patients with type 1 diabetes who received flexible PRO-based telehealth as part of their diabetes follow-up in Denmark, questioning whether this type of solution is acceptable for all patients. Our findings show that not all patients opt for digital outpatient care. A previous scoping review on reasons for nonuse of digital PRO identified factors, such as ability to use the PRO, engagement, emotional distress, and technical barriers [50]. Our identified differences in the use of insulin delivery equipment may represent differences in the technical skills required to use an insulin pump. Furthermore, the scoping review highlighted how participants with few symptoms or those in good health stopped using digital PRO solutions more frequently [50]. This aligns well with the differences in well-being among our groups, where those with higher well-being chose usual care.

In contrast to previous research highlighting the importance of socioeconomic background when offering digital health services, differences in such characteristics were not observed in our sample. Previous research highlights that health literacy and socioeconomically disadvantaged backgrounds may be less likely to be considered for digital PRO-based outpatient care [51]. Our findings show that there are no statistical differences in health literacy or socioeconomic status between the DigiDiaS care and usual care groups, suggesting that there is no reason not to consider such patients to use these solutions. However, this may be contextual, as the Norwegian population in general has a high socioeconomic status. Yet, further research can provide a deeper understanding of the specific characteristics that influence patients’ decisions to choose digital outpatient care.

Although we examined various characteristics from medical records and self-reported questionnaires, we did not investigate all potential influencing factors. For instance, while we assessed general health literacy to understand the ability to find, understand, evaluate, and apply health information, digital health literacy could provide deeper insights into patients’ ability to effectively use such solutions [52]. In addition, efforts should be made to explore whether the use of digital outpatient care solutions such as DigiDiaS care varies with factors, such as geography, travel time, and the availability of health care services. The introduction of new technology in health care services is a complex process [53], and patient acceptance of new solutions is influenced by how health care professionals adopt and support these technologies [54]. Further understanding and investigation of these dynamics may reveal factors for successful implementation in practice.

### Strengths and Limitations

This study presents strengths and limitations that are important to consider when interpreting the findings. First, this study explores a fully implemented digital outpatient care service [21], hindering the use of established gold standard research designs. However, our nonrandomized study design was essential in capturing the participants’ preferences, allowing them to choose either DigiDiaS care or continuing with usual care. This approach enabled us to investigate characteristics based on the participants’ choice of follow-up, which would not have been possible with other study designs, such as randomization or a stepped wedge design. The resulting skewed distribution was, therefore, expected and reflects these treatment choices. To compensate for the skewed distribution, nonparametric tests were used; nevertheless, the results should be interpreted with caution due to the uneven distribution.

While a none-randomized design with consecutive sampling may introduce some bias, it provides valuable insights into real-world decision-making and patient preferences that are crucial for understanding the adoption and effectiveness of the DigiDiaS care model [21], especially considering the needs and choices of the minority group who continued with usual care. Acknowledging that some participants do not wish to use this type of outpatient care is important, and the reasons for this should be further investigated. Qualitative interviews may reveal factors that we may not have detected in this study.

Second, this study does not examine the usability of individual components in the patient app or the specific solution of the app, but rather its clinical implications. Our investigation does not exhaustively cover all factors that may influence the choice of digital outpatient care given the complexity of such decisions. However, by collecting a broad range of data from medical records and self-reported data from the PRO questionnaire, we have gathered a comprehensive dataset. Our cross-sectional study, incorporating both generic and disease-specific well-validated measures, allowed us to add depth to the medical records data [55]. The richness of PRO data lies in the patient perspective it provides, reflecting their experiences, symptoms, and the impact of the disease on their daily lives [56].

Third, the digital outpatient care solution was introduced to half of the participants in the DigiDiaS care group before they were included in this study. The extent to which this was used is unknown, as its use is mainly related to the patients’ need to contact the clinic outside of fixed appointments, preparation for consultation or information, and e-learning courses received in connection with consultations. The time before we started recruiting for this study was also an introductory period in the use of DigiDiaS care for the clinic. We do not see it as a limitation that participants had access to the app before the start of the study, as we investigated opting for use of DigiDiaS care or not in this paper. However, as previously addressed, experiences from the Danish DiabetesFlex indicated that not everyone wanted to continue with DiabetesFlex after the study period [17], and even though our participants were opting for DigiDiaS care, actual use should be further investigated.

### Conclusions

This study reveals that patients with type 1 diabetes are opting to use a digital outpatient care solution when it is offered. Most of participants opted for DigiDiaS care, the novel mHealth solution, while a smaller proportion opted to continue with usual care. Those opting for DigiDiaS care are characterized by being already familiar with using diabetes-related technology, having a shorter diabetes duration and lower well-being than the usual care group.

Importantly, our findings indicate that factors such as demographic and clinical variables, as well as self-management, health literacy, and diabetes distress did not differ significantly between DigiDiaS care and usual care. Even though only a small proportion continued with usual care, the characteristics of this group should be further explored to understand how these individuals can also be included in digital outpatient care solutions. Further studies should also focus on how the implementation of digital solutions in outpatient care can affect how and if patients use them.

## Abbreviations

HbA1c: glycated hemoglobin
mHealth: mobile health
OR: odds ratio
PAID-20: 20-item Problem Area in Diabetes
PAM-13: Patient Activation Measure 13
PRO: patient-reported outcome
STROBE: Strengthening the Reporting of Observational studies in Epidemiology
HLS19-Q12: 12-item short version of the European Health Literacy Survey Questionnaire
WHO-5: World Health Organization-Five Well-Being Index

## Data Availability

The datasets generated or analyzed during this study are available from the corresponding author upon reasonable request.
